# Functional Data Analysis for the Structural, Chemical, Thermal, and Mechanical Properties of PA12 Additively Manufactured via SLS

**DOI:** 10.3390/polym17202763

**Published:** 2025-10-15

**Authors:** Alejandro García Rodríguez, Yamid Gonzalo Reyes, Edgar Espejo Mora, Carlos Alberto Narváez Tovar, Marco Antonio Velasco Peña

**Affiliations:** 1 Facultad de Mecánica, Escuela Tecnológica Instituto Técnico Central, Calle 13 No. 16-74, Bogotá 111411, Colombia; 2 Facultad de Ingeniería Mecánica, Universidad Santo Tomás, Carrera 9 No 51-11, Bogotá 110231, Colombia; yamid.reyes@usta.edu.co; 3 Departamento de Ingeniería Mecánica y Mecatrónica, Universidad Nacional de Colombia, Carrera 45 No. 26-85, Bogotá 111321, Colombia; eespejom@unal.edu.co (E.E.M.); canarvaezt@unal.edu.co (C.A.N.T.); 4 Facultad Tecnológica, Universidad Distrital Francisco José de Caldas, Calle 13 No. 31-75, Bogotá 110231, Colombia; mavelascop@udistrital.edu.co

**Keywords:** FDA (functional data analysis), PA12 (polyamide 12), material properties

## Abstract

Additive manufacturing via selective laser sintering (SLS) enables the rapid production of geometrically complex polyamide 12 (PA12) components. However, conventional pointwise analysis techniques often overlook the full depth of continuous experimental datasets, thus limiting the interpretation of structure–function relationships that are essential to high-performance design. This study employs functional data analysis (FDA) to elucidate the microstructural, chemical, thermal, and mechanical behaviours of SLS-fabricated PA12, focusing on the effects of build orientation (horizontal, transverse, vertical) and wall thickness (2.0–3.0 mm). The samples were produced via a commercial SLS platform and characterised via X-ray diffraction (XRD), Fourier transform infrared spectroscopy (FTIR), differential scanning calorimetry (DSC), and tensile testing. The FDA was applied to raw, normalised, and first derivative datasets via Python’s Scikit-FDA package, increasing the sensitivity to latent material variations. The findings demonstrate that the build orientation has a marked influence on the crystallinity and mechanical performance: horizontal builds yield narrower gamma-phase XRD peaks, greater structural order, and enhanced tensile properties, whereas vertical builds exhibit broader peak dispersion and greater thermal sensitivity. The wall thickness effects were minor, with only isolated flux-related anomalies. The FTIR spectra confirmed the consistent chemical stability across all the conditions. The FDA successfully identified subtle transitions and anisotropies that eluded traditional methods, underscoring its methodological strength for advanced polymer characterisation. These insights offer practical guidance for refining SLS process parameters and improving predictive design strategies in polymer-based additive manufacturing.

## 1. Introduction

Additive manufacturing (AM) has emerged as a rapidly expanding and transformative technology in modern engineering, offering solutions beyond the reach of conventional fabrication methods [1,2,3]. Through various AM techniques, parts with high geometric complexity can be produced with improved material efficiency and reduced environmental impact [4]. Among these techniques, selective laser sintering (SLS) stands out for its sustainability benefits, including the use of recycled materials and the ability to fabricate self-supporting components that minimise material waste [5,6,7,8,9]. In addition, SLS enables precise control of anisotropic behaviour when the material composition, chamber conditions, and laser energy input are finely managed [10,11,12].

Extensive research has explored how SLS process parameters influence the mechanical, chemical, surface, and structural properties of fabricated components, particularly polyamide 12 (PA12) [13,14,15,16,17]. Characterisation methods such as Fourier transform infrared spectroscopy (FTIR), X-ray diffraction (XRD), and differential scanning calorimetry (DSC) are widely applied. However, their analyses are often limited to pointwise data—isolated values such as stress peaks [18], functional group wavelengths [19], diffraction angles [20,21,22], or thermal transitions [23]. This approach typically represents less than 1% of the available dataset, leaving most of the continuous information unexamined. As a result, the rich data captured during testing remain underutilised, and subtle patterns in behaviour are often overlooked.

Functional data analysis (FDA) is an emerging statistical technique that can interpret entire data curves across a domain—such as time, temperature, or deformation—concerning input parameters [24,25,26,27]. The FDA utilises statistical tools, including descriptive analysis, ANOVA, post hoc testing, and entropy measures, to detect differences that traditional pointwise analyses may overlook. However, while the FDA has proven effective in diverse engineering contexts, its use in additive manufacturing remains limited. Existing studies largely restrict the FDA to mechanical testing with uniform datasets, overlooking its applicability to other techniques, such as FTIR, XRD, and DSC. Stress–strain curves often contain varying numbers of data points due to differing test conditions. This variability poses challenges for conventional FDA applications. Although domain equalisation offers a potential solution by aligning heterogeneous datasets into comparable domains, this approach is still underutilised in the field [28].

FDAs present significant advantages for additive manufacturing, especially in studies constrained by limited access to characterisation techniques or resources. Through normalisation, derivative enhancement, and domain-specific metrics—including diffraction angles, wavelengths, and thermal profiles—it becomes possible to reveal subtle material differences that standard analysis methods may miss. Given the complexity of the response of PA12 to sintering variables, the FDA offers a robust framework for examining orientation-dependent behaviour and wall thickness sensitivity.

Therefore, the present study applies the FDA to comprehensively characterise the microstructural, chemical, thermal, and mechanical properties of PA12 components fabricated via SLS. It aims to evaluate and quantify functional differences in the build direction—horizontal, transverse, and vertical—and wall thickness conditions, using raw, normalised, and derivative data curves. Statistical tools, including descriptive analysis, ANOVA, and HIC tests, are employed to assess variations, whereas entropy analysis is conducted to examine homogeneity within the functional data. This methodology enables a nuanced understanding of interdependent factors in SLS-manufactured PAs12 and proposes the FDA as a robust tool for uncovering complex material behaviours.

## 2. Materials and Methods

### 2.1. Specimen Manufacturing Conditions

Uniaxial tensile samples were fabricated on a commercial EOS Formiga P110 Velocid machine (EOS GmbH, Munich, Germany) according to the manufacturer’s recommended criteria, following ISO 527-Type I guidelines [29]. The samples were manufactured via a CO_2_ laser with a power of 30 W, a scan speed of 5 m/s, a layer thickness of 0.12 mm, and a build rate of 1.2 L/h. An EOS polyamide 12 mixture of virgin powder and used powder (30:70) was used, and the machine predetermined the scanning strategy.

### 2.2. Experimental Design and Statistical Analyses

The experimental design involves two variables, each with three levels. The first variable is the direction of the building, which can be categorised into horizontal, transverse, and vertical levels. The second variable is the wall thickness, with values of 2.0, 2.5, and 3.0 mm. For the chemical, structural, and thermal tests, there is one result per condition, for a total of nine results. For mechanical characterisation, eight replicates per condition were used, resulting in 72 curves.

### 2.3. Transform Fourier Infrared (FTIR) Curves

Infrared spectroscopy (FTIR) spectra were obtained via attenuated total reflection (ATR) via a Shimadzu IRAffinity-1 s spectrophotometer (Kyoto, Japan). The measurement conditions were a spectral resolution of 8 cm^−1^, 64 scans per spectrum, and a wavenumber range of 200–4000 cm^−1^.

### 2.4. X-Ray Diffraction (XRD) Curves

The powder crystalline structure was determined via a Philips diffractometer (Eindhoven, The Netherlands) with a 30 kV voltage and 20 mA current operating in the Bragg–Brentano configuration with Kα signals from the copper anode (λ = 0.1542 nm), 2θ values between 5° and 50°, and a step size of 0.02°.

### 2.5. Differential Scanning Calorimetry (DSC) Curves

A Mettler Toledo differential scanning calorimeter (1–500/227), Alemania, Gießen, Germany, was used for thermal characterisation. The temperature range was between 25 and 300 °C, with a heating and cooling rate of 10 °C/min in a N_2_ atmosphere.

### 2.6. Stress–Strain Curves

The stress–strain curves were taken on a Shimadzu universal testing machine AG IS-5KN with an SLBL-5K load cell capacity of 5 kN. The test speed was 2 mm/min. The strain was measured via a Shimadzu contact extensometer, SES 1000, equipped with a 25 mm gauge that was adapted to the extensometer.

### 2.7. Domain Discretisation

The curves associated with the FTIR, DSC, and XRD techniques have the same number of points between tests, so the grid domain is associated with the independent variable. In FTIR, it is the wavelength; in DSC tests, it is the temperature; and in XRD, it is the diffraction angle. The curves associated with the mechanical test exhibit different domains, so we proceed to project each variable onto a grid with a specified number of points. A linear interpolation was performed using the vector with the highest number of points to equalise the domains.

### 2.8. Functional Data Analysis

The functional data analysis algorithms were performed in Python v3.13.7 via the Scikit-FDA library. DSC, XRD, and FTIR curves were analysed via normalisation. Owing to the semiquantitative nature of the technique, the transmittance level, heat flux, and transmittance percentage were normalised. For each of the characterisation tests, we analysed the raw curve obtained from the study and the first derivative.

## 3. Results and Discussion

### 3.1. X-Ray Diffraction (XRD)

**Figure 1:**
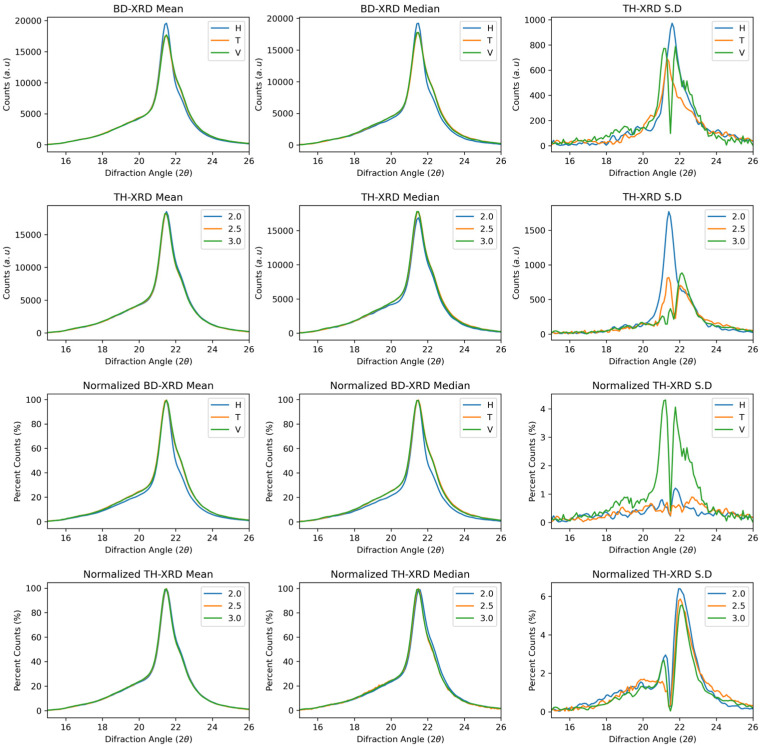
XRD spectra of PA12 samples showing phase signals across build directions and wall thicknesses. First and second rows: Raw spectra. Third and fourth rows: Normalised spectra.

Figure 1 shows the median, mean, and standard deviation curves as a function of wall thickness and build direction for the raw data with and without normalisation. The diffractogram shows a signal corresponding to the Gamma phase of the polyimide 12, associated with the material’s rapid cooling after the laser passage [30,31]. The Gamma phase crystal shows a preferential parallel orientation with helical conformation chains [32]. Two shoulders are observed, one below the gamma signal (<21°) and one after the signal (<21°), corresponding to the alpha phase, which is characteristic of slow cooling [33]. The orientation of the crystals is antiparallel to the stretching of the chains. The peak presents a robust base related to the semicrystalline condition of the material [20,21].

In the raw and normalised curves, the gamma phase was identified as predominant regardless of the build direction and wall thickness. This behaviour would be natural since cooling after the laser passes through would occur within seconds, favouring the growth of the gamma crystals. Despite this, the mean and median curves in the horizontal direction presented a greater number of counts in the primary gamma signal. In addition, the horizontal signal has a smaller peak width (22% lower than the transverse peak width and 26% lower than the vertical peak width), which may be related to an increase in the crystallinity of the sample [34]. The vertical and transverse directions exhibit an increase in the alpha signal compared with the horizontal direction in both the raw and normalised curves. Compared with those in the horizontal direction, the sintering areas in the transverse and vertical directions are 40% and 75% lower, respectively. Small sintering areas may experience thermal overload. This leads to slower cooling from heat accumulation, which in turn increases the peak width in the alpha phase [34,35].

On the other hand, despite their relatively slow cooling compared with the horizontal direction, the cooling time of these parts is in seconds, so there would be a greater proportion of gamma–alpha phase combinations and therefore a greater dispersion of crystal size, which would explain the increase in peak width [36]. This behaviour is present in both the raw curves and the normalised curves, where it is most noticeable in the normalised peak width, highlighting changes in the crystal structure in the horizontal direction. The curves associated with wall thickness did not show significant changes in either the raw or normalised curves.

The functional standard deviation supports the above hypothesis, as the highest dispersion before and after normalisation in the horizontal direction is observed in the signal corresponding only to the gamma phase. As the sintering area decreases, the deviation increases at the gamma–alpha interface, indicating greater dispersion in the transverse direction, followed by the vertical direction.

**Figure 2:**
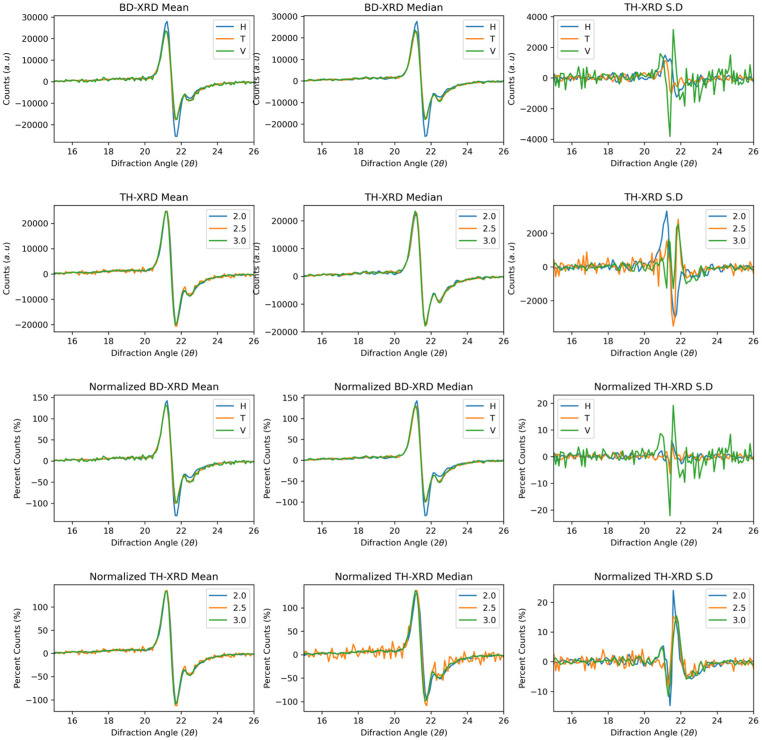
First derivative XRD curves showing gamma and alpha phase transitions. Third and fourth rows: First derivative Normalised spectra.

Figure 2 shows the derivative of the XRD functions as a function of the build direction and wall thickness. The first derivative allows the Gamma and Alpha phase signals to be distinguished more clearly. The gamma phase at the 21° angle shows two peaks, each with a positive value representing the first half of the peak and a negative value representing the right half. A distinguishable signal with a reduced intensity near 22° represents the alpha phase of polyamide 12. This behaviour was present in the curves as a function of the build direction and wall thickness. As a function of the build direction, both with and without normalisation, the horizontal direction presented a greater intensity in terms of counts and percentage, respectively, than did the vertical and transverse directions. In the normalised curves, the change is more noticeable in the curve at negative values, indicating that the diffraction curve lies more towards higher angle values. This tendency may be due to two factors: residual stresses, crystal size gradients, and signal overlap, as the horizontal direction has a relatively high cooling rate. Aupt cooling could help mitigate internal stresses that contribute to the asymmetry of the peak [33,37,38].

On the other hand, gradient crystal sizes towards higher angles of the gamma phase or the mixing of gamma and alpha crystals could explain the signal asymmetry [34]. On the other hand, the right shoulder associated with the Alpha phase is stronger in the transverse and vertical directions in the raw and normalised spectra. Like the gamma phase, there are two peaks associated with each half of the signal dome. The signal in the horizontal direction tends to have more linear behaviour, with symmetry between the positive and negative parts. This symmetry confirms the reduction in the Alpha phase under these conditions.

Conversely, in the transverse and vertical directions, the signal is stronger and more symmetrical, indicating that the alpha phase is present in greater quantity in these two directions. The curves associated with the wall thickness, as in the initial condition, did not present a significant difference in the signal. Notably, the XRD curves associated with a 2.5 μm thick sample exhibit greater background noise; however, the signal in the central peak is clear. For the standard deviation, the derivative is more sensitive to minor changes in the function’s behaviour, which triggers the signal in the alpha and gamma phase zones. This behaviour is more erratic in the curves of the build directions. The thickness presents a slightly more uniform behaviour; however, the white noise of the signal affects this functional statistic.

**Figure 3:**
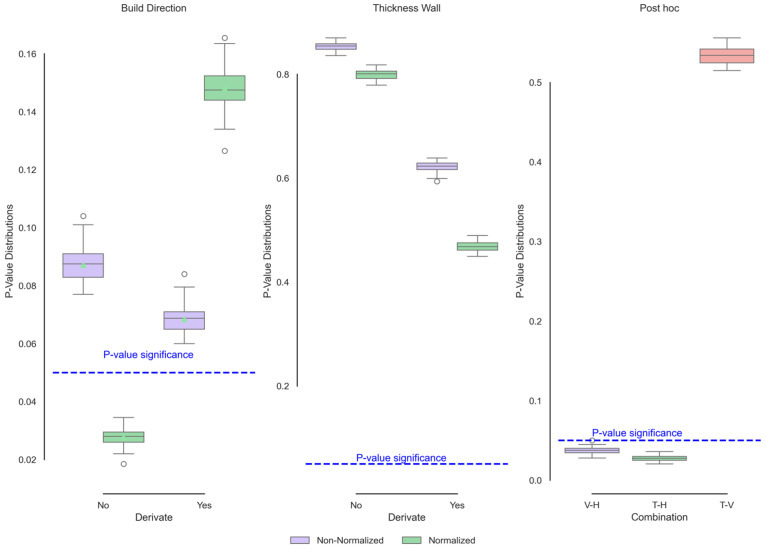
Functional ANOVA of XRD data showing significant build direction effects post-normalisation.

Figure 3 (left and centre) shows the iterated *p* values of the XRD curves as a function of wall thickness and the curves of comparison between means (post hoc-right tests) of the coding that showed significant differences. The wall thickness, as described by its mean, median, and functional standard deviation behaviours above, clearly did not present significant differences, so this variable would not affect the behaviour of the atomic structure. On the other hand, only significant functional differences were observed in the build direction subjected to the normalisation process. By normalising the curves, the differences corresponding to the “beads” are avoided. The intensity of the beads, which depends on the sample’s mass and porosity, can cause changes in the intensity ratio received by the sample. By normalising the samples, the properties that affect the emission intensity are reduced, and the curve can be focused on movements in the diffraction angles or changes in the peak width. It is clear from Figure 3 that the horizontal direction shows a decrease in the peak width of the Gamma signal. In addition, the shoulder corresponding to the Alpha phase decreases in size, indicating greater crystalline ordering and/or a reduction in the crystal size.

### 3.2. Spectrocospy Infrared Transmittance (FTIR-ATR)

**Figure 4:**
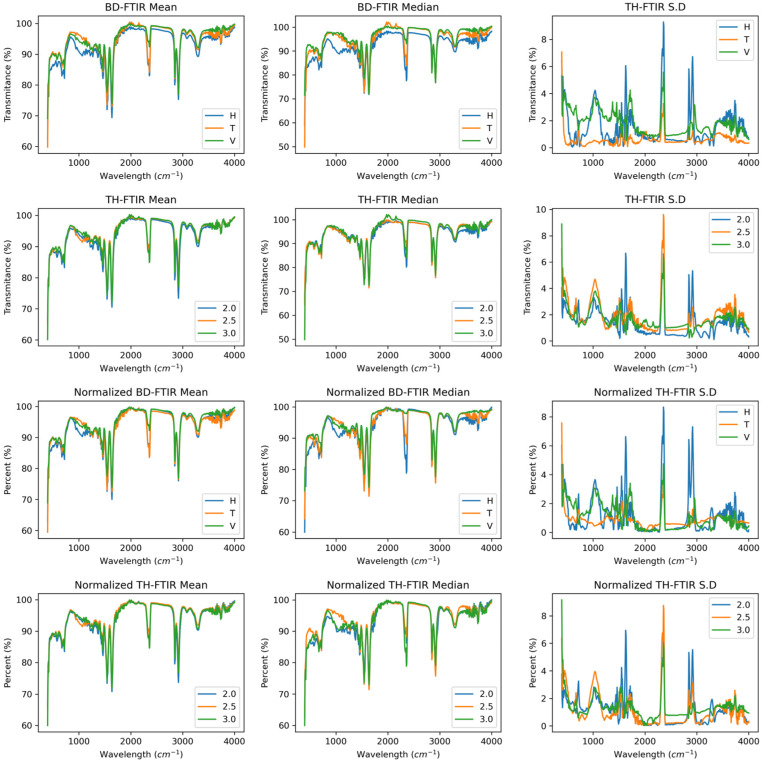
FTIR spectra of PA12 samples showing consistent functional group signals across conditions. First and second rows: Raw spectra. Third and fourth rows: Normalised spectra.

Figure 4 shows the median, mean, and standard deviation curves as a function of wall thickness and building direction, illustrating the chemical behaviour of Polyamide 12 sintered by SLS. Infrared spectral changes in transmittance occur in all directions, as shown in the mean and median curves; however, the changes are less than 10% in the functional groups. Normalising the curves as a function of the build direction, it was observed that the transmittance dispersion tends to decrease, exhibiting the same behaviour in all directions. The standard deviation indicates that at 1200, 2500, and 3000, the highest variability of the data is observed, corresponding to the amide III, CH_2_, and N–H groups [39]. For the wall thickness, less dispersion was observed in the chemical behaviour direction than in the build direction. When the IR curves are normalised, the dispersion tends to decrease, indicating more homogeneous behaviour.

**Figure 5:**
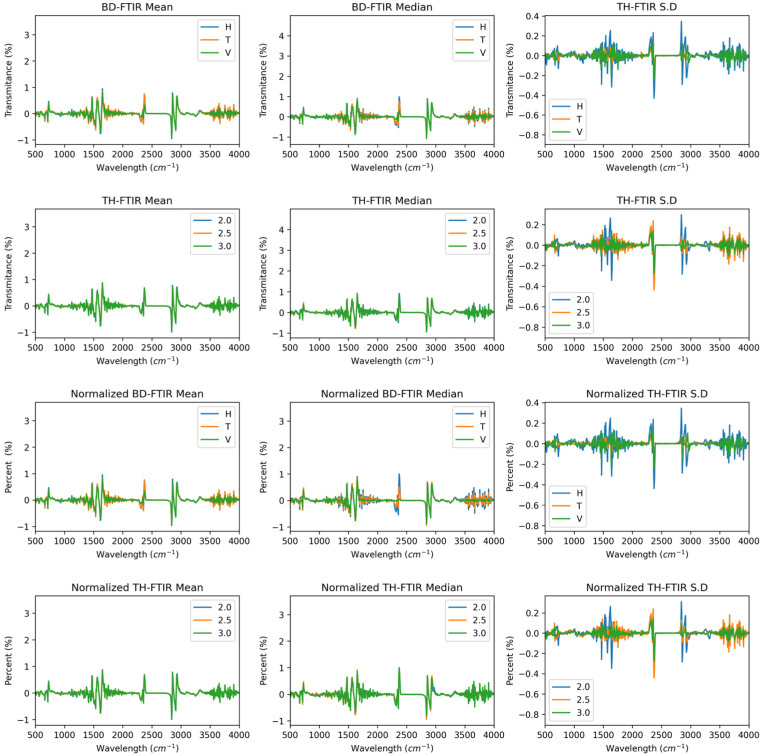
Derivative FTIR curves revealing intensity shifts near 2350 cm^−1^. First and second rows: First derivative raw spectra. Third and fourth rows: Second derivative normalised spectra.

Figure 5 shows the derivatives of the FTIR curves corresponding to the spectra as a function of the build direction and wall thickness. The directions of the signals corresponding to the functional groups did not notably differ between conditions, except for the signal near 2350, which corresponds to the O=C=O group [19]. This phenomenon is further enhanced by normalising the curves, which shows that the horizontal and transverse directions exhibit an increase in this signal. On the other hand, the wall thickness and the derived spectra appear to be homogeneous under all conditions, both with and without normalisation of the data.

**Figure 6:**
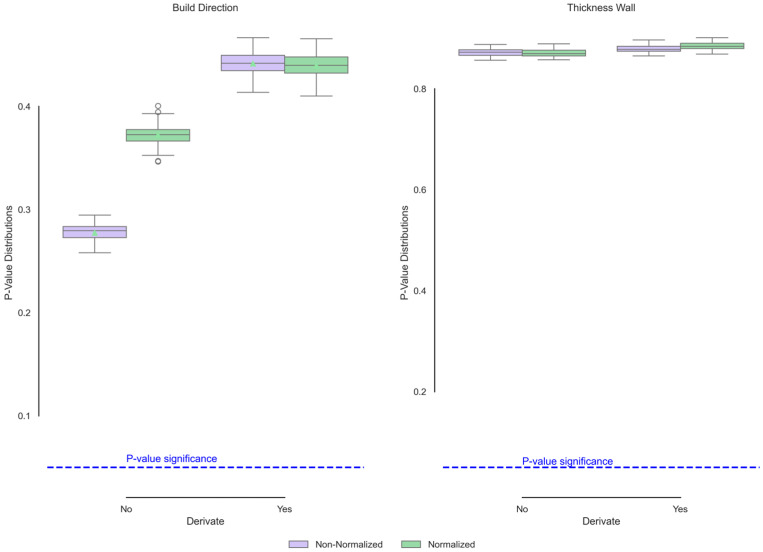
Functional ANOVA of FTIR data confirms no significant chemical differences.

Figure 6 shows the iterated *p* values of the FTIR curves as a function of wall thickness and build direction. None of the conditions presented significant changes in the chemical composition based on the input variables. The samples are manufactured in the same production batch via the same laser and mixing conditions; these conditions do not affect the functional groups of polyamides 12 [40]. Although studies such as Anjos et al. [41] have shown that the derivative of the spectra in IR transmittance is more sensitive to change, in this case, the *p* value indicates that the input variables have no effect on this measure, which is much more sensitive to change. These *p* values suggest that the observed changes in the build direction may be attributed to other factors, such as sample preparation, internal porosity, sample thickness, or conditions related to equipment operation [42]. Furthermore, the thickness is not sensitive to these factors because of the homogeneity of the *p* values.

### 3.3. Differential Scanning Calorimetry (DSC)

#### 3.3.1. Exothermic Behaviour

**Figure 7:**
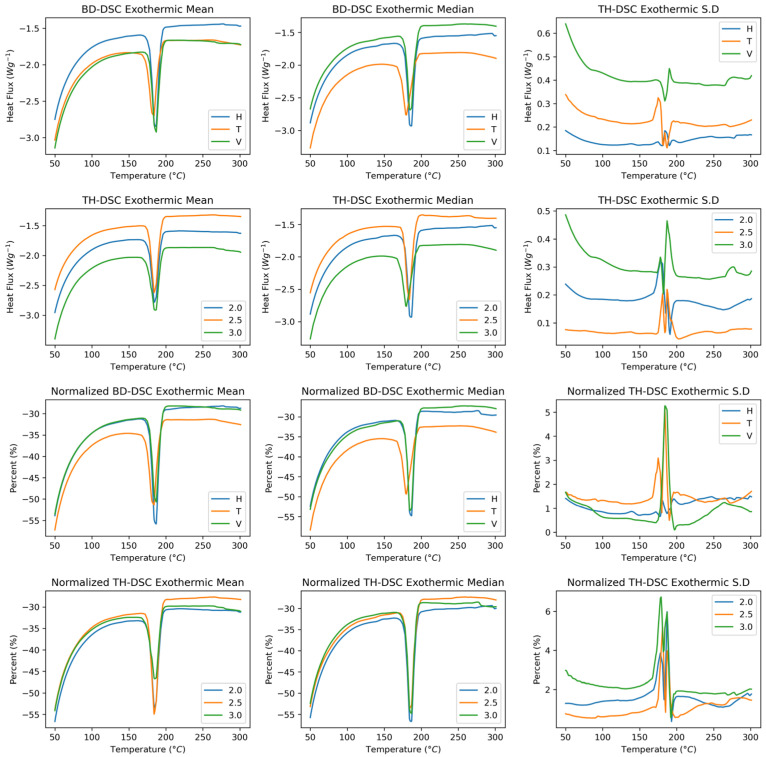
Exothermic DSC curves of PA12 samples as a function of build direction and wall thickness. Raw and normalised heat flux profiles shown. First and second rows: Raw spectra. Third and fourth rows: Normalised spectra.

Figure 7 shows the exothermic behaviour of the SLS-manufactured polyamide 12 samples as a function of the build direction and wall thickness. The build direction is identified as having changes in both the heat flux and the temperature to which the sample is subjected. The mean exothermic behaviour curve shows greater closeness between the horizontal and transverse conditions. The standard deviation shows that the direction with the most significant standard deviation is vertical, as with the other characterisation techniques. With respect to wall thickness, changes were primarily observed in the sample heat flux, which exhibited similar behaviour between thicknesses of 2.5 mm and 3.0 mm. The standard deviation increases with increasing thickness. When normalisation is applied to the exothermic components as a function of the build direction, the vertical and horizontal directions exhibit similar behaviours; however, in the melting peak region, the horizontal and vertical directions shift towards higher fusion temperatures. This behaviour is associated with a change in the standard deviation, with an increase of up to 5% in the data deviation in the vertical direction. With respect to the normalised wall thickness, homogeneous mass behaviour is observed for the temperature variable; however, the signal intensity at a thickness of 3.0 mm is greater. The standard deviation presents similar behaviours greater than 6% of the data.

**Figure 8:**
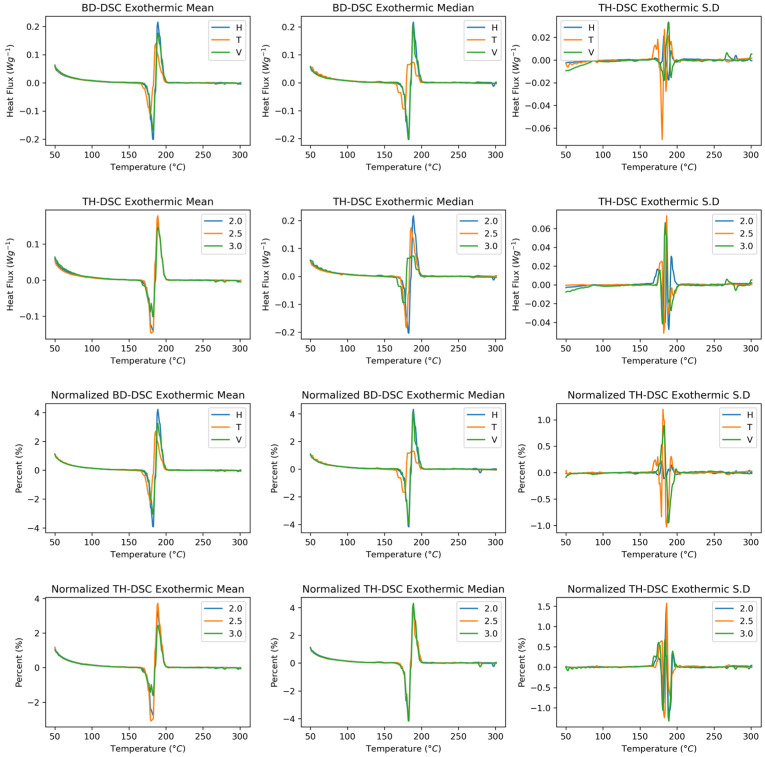
First derivative of exothermic DSC curves. First and second rows: First derivative raw spectra. Third and fourth rows: First derivative normalised spectra.

Figure 8 shows the derivative of the exothermic behaviour of the samples manufactured in Polyamide 12 by SLS. The curves associated with endothermic behaviour as a function of the build direction show more notable changes in the displacement of the fusion temperature, with the vertical and horizontal directions presenting the highest values. In terms of the heat flux, the transverse mean and median curves are lower than those under the other conditions. Concerning the standard deviation, the fusion zone exhibits more notable changes in the transverse direction than in the other two directions. Compared with the other two wall thicknesses, the 2.5 mm thick wall presented changes in heat flux. No notable changes were observed in the shifts at the fusion temperatures. In this case, the 3.0 wall thickness presents the most significant data dispersion. When the data are normalised, the behaviour is practically the same in the build direction, showing variations of up to 1% standard deviation in the fusion zone. A homogenisation of the wall thickness results is observed; however, the average curve shows that a thickness of 2.5 mm is the most intense.

**Figure 9:**
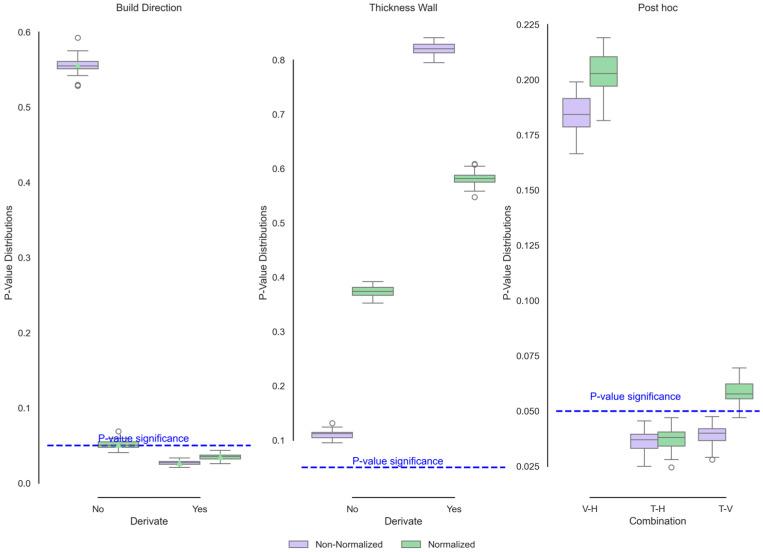
Functional ANOVA of exothermic DSC data as a function of direction of buildup and wall thickness showing significant differences in build direction linked to porosity and crystallinity.

Figure 9 shows the functional ANOVA and post hoc group comparisons of the exothermic behaviour of the polyamide 12 samples manufactured by SLS. The wall thickness, as determined by X-ray diffraction (XRD), does not significantly differ in function. The differences in thickness behaviour can be attributed to the test mass. Although the thickness is a design condition, it varies in each direction, and in the vertical direction, it exhibits the greatest dispersion. This variation would indicate and explain why the conditions change between them.

On the other hand, it was evident that the build direction presented changes in the derivative. The derivative of a function results in changes in the signals that cannot be observed in raw conditions. These differences were distinguished between the transverse condition and the other two conditions because of the percentage of porosity and the air mass ratio. Similar studies have shown that the vertical and horizontal directions present a greater percentage of internal porosity, which would explain the difference from the vertical direction as a function of heat flow and temperature. When normalised, this effect of internal porosity is reduced, indicating that the only difference is between the horizontal and transverse conditions. In this case, the difference would only be associated with the melting temperature. On the basis of the XRD findings and the area under the DSC curve, the horizontal direction yields a greater degree of crystallinity, followed by the vertical direction and, finally, the transverse direction. Therefore, the shifts towards higher temperatures are significant since a greater amount of energy is required for the change in the state of matter.

#### 3.3.2. Endothermic Behaviour

**Figure 10:**
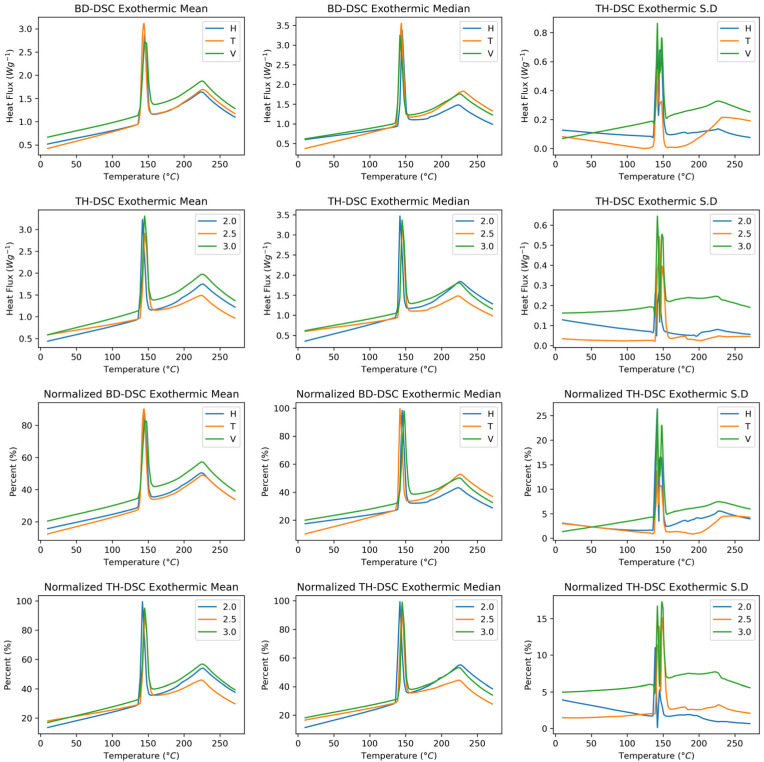
Endothermic DSC curves showing crystallisation behaviour of PA12. Raw and normalised data across build directions and wall thicknesses. Vertical builds exhibit higher heat flux. First and second rows: Raw spectra. Third and fourth rows: Normalised spectra.

Figure 10 shows the endothermic behaviour of the SLS-manufactured polyamide 12 samples as a function of the build direction and wall thickness. The crystallisation peak is identified in the signal, corresponding to the temperature at which the semicrystalline polymer begins to generate chain order and therefore crystal formation. Analysing the behaviour as a function of build direction, the base signal of the functions tends to change; however, at the crystallisation peak, all directions present homogeneous behaviour. An increase in heat flux is highlighted in the signal corresponding to the transverse direction in the mean and median curves. The functional standard deviation indicates that the heat flux varies the most in the vertical direction and that the baselines exhibit a notable trend.

With respect to wall thickness, a similar behaviour to that observed in the build direction is found in the mean, median, and standard deviation functional statistics. When the samples are normalised, changes are observed in the mean curves, where the vertical condition presents greater heat fluxes. Despite this, the median curve shows homogeneity between the heat fluxes. The exothermic curves, as a function of thickness, exhibit homogeneous behaviour in terms of heat fluxes and shift from higher thickness values to higher crystallisation temperatures.

**Figure 11:**
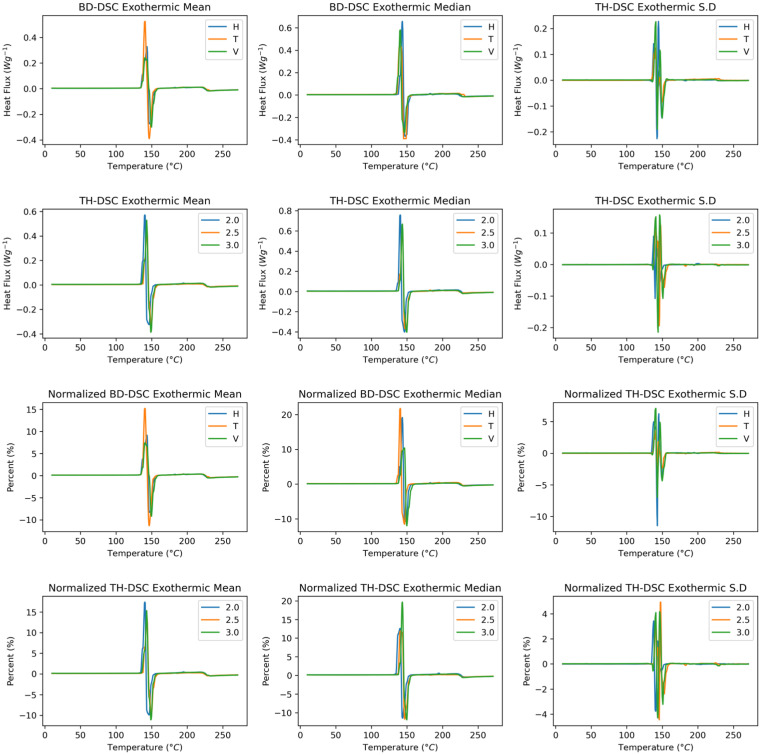
First derivative of endothermic DSC curves showing dispersion in crystallisation peak. First and second rows: First derivative raw spectra. Third and fourth rows: First derivative normalised spectra.

Figure 11 shows the derivative of the endothermic behaviour of the SLS-manufactured samples as a function of the build direction and wall thickness. The curves versus the build direction show, as in the rough curves, that the vertical direction results in the most significant heat flux. This behaviour is less noticeable in the mean curve. This finding in the derivative and rough curves indicates a high dispersion of the curves, where the mean curve is generally more sensitive to changes in extreme conditions than the mean curve is. This behaviour is replicated in both the wall thickness direction and the build direction, with and without normalisation. The standard deviation is most dispersed at the peak, with the transverse direction exhibiting the most significant variation in the data.

**Figure 12:**
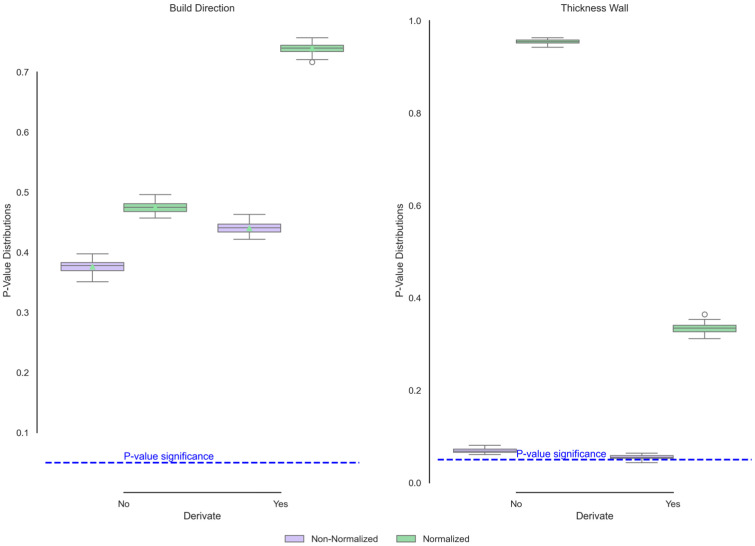
Functional ANOVA of endothermic DSC curves showing no significant variation in thermal response.

Figure 12 illustrates the functional ANOVA and group comparisons of endothermic behaviours as a function of build direction and wall thickness. Notably, in this case, the build direction and wall thickness do not result in significant changes in the crystallisation temperature. These changes can be attributed to the fact that, in the DSC test, endothermic behaviour is observed after exothermic behaviour, resulting from the slow cooling of the material. In this case, the sample starts as a molten material and cools gradually, so the initial conditions would not initially have an influence. For wall thickness, the non-normalised *p* values present significant differences; however, these differences are not associated with temperature but rather with heat flux. By having different mass conditions, the transfer would change, which would explain the proximity to the significance limit. Even without normalisation, most *p* values remain above 0.05. Only a few range values approach significance, but they do not fall within the first to third quartiles.

### 3.4. Mechanical Properties

**Figure 13:**
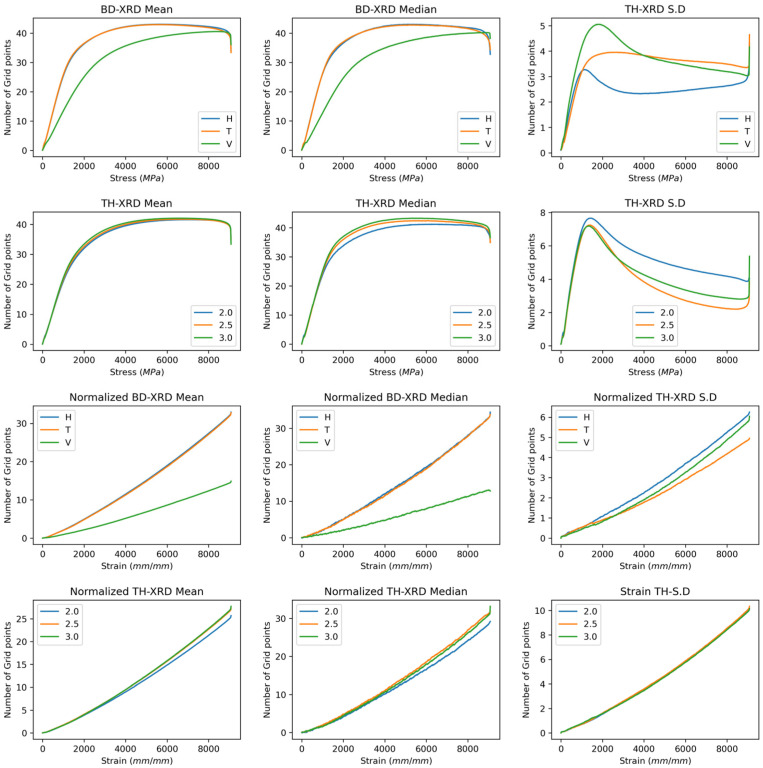
Stress–strain curves indicating reduced mechanical resistance in vertically built samples. First and second rows: Raw stress spectra. Third and fourth rows: Strain-normalised spectrum.

Figure 13 shows the functional curves of the stress and strain in the raw state as a function of the build direction and wall thickness. The stress, as a function of the build direction, exhibited very marked behaviour when analysing the transverse and horizontal directions against the vertical direction. The median and mean curves indicate a decrease in stress in this section as the elastic zone transitions to the plastic zone, confirming the low resistance to ultimate stress. Variations of up to 5 MPa were observed in the transition zone between the elastic and plastic zones. Owing to the elastic–plastic regime, this zone can vary between different manufacturing conditions, which explains variations of up to 10%. There is then an increase in the rupture zone; however, this is expected, as the rupture stress is multifactorial and tends to exhibit considerable variability. The mean and median curves are quite similar, with the highest thickness resulting in the highest resistance. With respect to the direction of impression, the functional deviation shows the most significant variation at the elastic–plastic interface; however, in this zone, it decreases, indicating much more homogeneous behaviour. The deformation behaviour was similar to that of the stress in both the build direction and the wall thickness. The vertical direction presented the lowest mean and median curves of the conditions, clearly differentiating the behaviour. The standard deviation shows an increase; this can be attributed to the fact that plastic deformation varies between conditions. Despite this, the functional deviation curves are similar, indicating a certain degree of homogeneity in each of the tests—deformation as a function of wall thickness. Notably, the mean, median, and standard deviation curves are very similar, indicating less variability in this factor.

**Figure 14:**
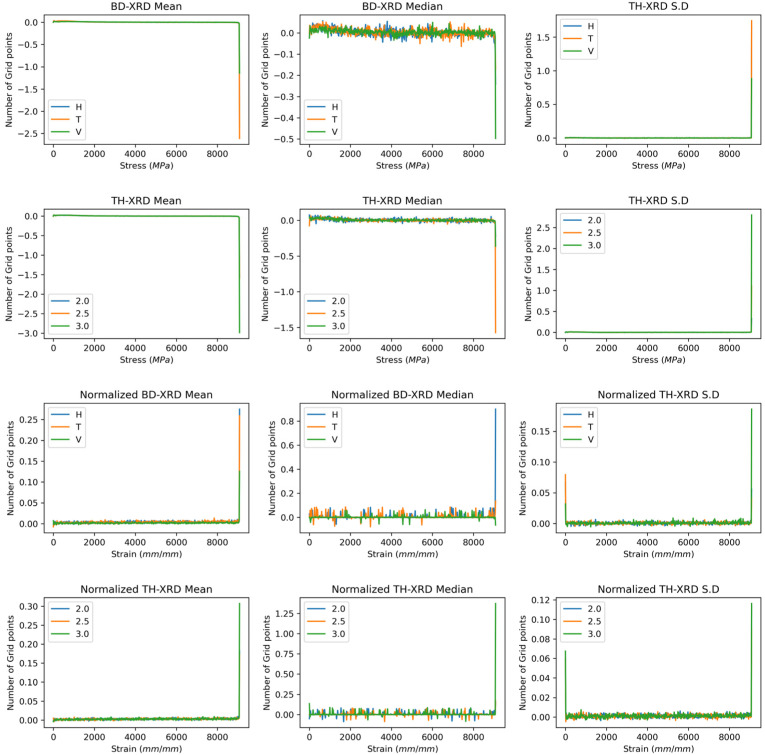
First derivative of stress curves showing rupture zone transitions across build directions. First and second rows: stress derivative raw spectra. Third and fourth rows: Strain derivative normalised spectra.

Figure 14 shows the first derivatives of the stress and strain functions as a function of the build direction and wall thickness. Under all conditions, the function, being a smooth curve, is sensitive only to the abrupt variability of the data, i.e., the breakage zone shows an increase in the signal. This variability would indicate that in curves that do not exhibit drops in behaviour, the first derivative is not sensitive, as noticeable changes only occur at the breakage of the material.

**Figure 15:**
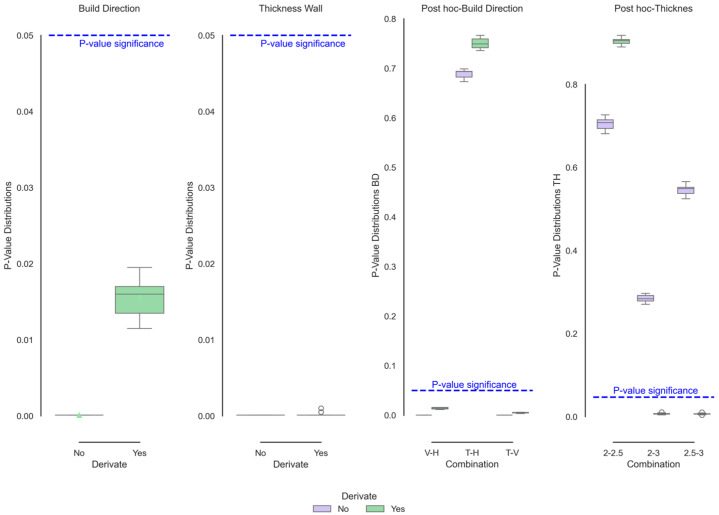
Functional ANOVA of stress data revealing significant differences attributable to build orientation.

Figure 15 shows the post hoc tests corresponding to the functional ANOVA of the stress as a function of the build direction and wall thickness. Although the first derivative visually shows no apparent change, both this dataset and the raw data are sensitive to the build directions. As indicated in the raw data, a noticeable decrease in stress in the vertical direction, indicating lower resistance in the elastic and plastic zones. There was no difference between the horizontal and transverse directions, indicating that the stresses are similar in each region of the material. For the wall thickness, although the difference is minimal in the raw curves, it becomes noticeable in the derivative curves, where a distinction is evident between the minimum and maximum thicknesses. This change indicates that the sensitivity of the curve changes depending on the treatment. In the raw curves, the thickness is neglected; however, with the derivative, it resembles the singular difference found punctually in this parameter.

**Figure 16:**
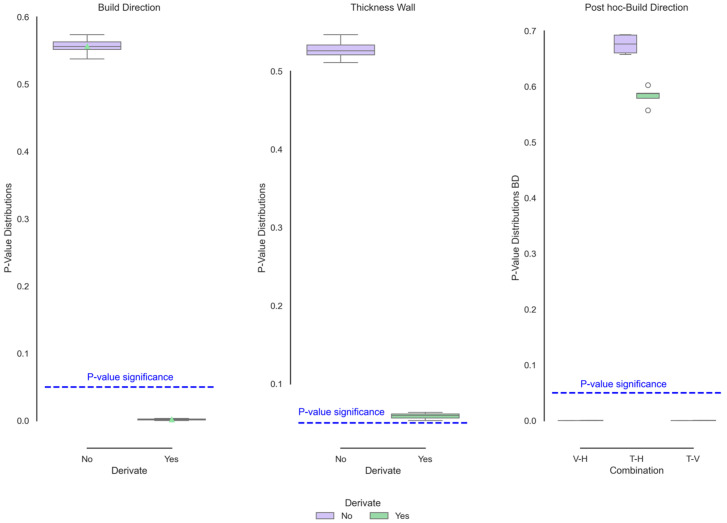
Functional ANOVA of strain data showing lower strain values in vertically fabricated.

Figure 16 shows the post hoc tests corresponding to the functional ANOVA of the deformation as a function of the build direction and wall thickness. In contrast to the stress, only the direction significantly changed in this section. The vertical direction clearly showed significant changes towards lower strain values. In contrast, the wall thickness, conversely, showed such slight differences that the derivative could not be determined.

According to the punctual analysis carried out by Rodriguez et al. [40,42] and compared with the present research, the derivative is closer to the actual condition of the stresses, taking the wall thickness and the direction of construction as variables that present significant changes. This behaviour can be linked to the thermal and structural samples, where the direction was the variable that presented the most significant changes in function. Since the mechanical strength is linked to the cooling and phase generation factors, the mechanical behaviour of the stress found is congruent with its physico-chemical properties. This difference is mainly between the vertical and transverse directions. On the other hand, although the derivative presents *p* values close to the limit of significance, the thickness does not present a significant difference in deformation, with the direction of impression being the most important criterion for both mechanical and physical–chemical behaviours.

## 4. Conclusions

This study employed functional data analysis (FDA) to comprehensively characterise polyamide 12 (PA12) components fabricated via selective laser sintering (SLS) and examined how build direction and wall thickness influence structural, chemical, thermal, and mechanical properties. Unlike traditional pointwise approaches, the FDA’s capacity to analyse entire data curves proved essential in revealing nuanced material behaviours, which are often imperceptible through conventional metrics. Notably, the build direction was found to affect the internal crystalline structure significantly, dictating phase formation and crystallinity levels, as well as thermal responses such as melting and crystallisation temperatures.

Mechanical tests revealed that horizontally and transversely built parts consistently outperformed their vertically built counterparts in terms of strength and resistance. In contrast, chemical stability remained remarkably consistent across orientations and thicknesses. These insights hold practical significance for professionals engaged in SLS additive manufacturing, as a clearer understanding of how build parameters influence the final properties of PA12 parts enables more informed design choices, enhanced process control, and improved predictability of part performance.

Beyond characterisation, the results directly inform SLS process optimisation. The FDA’s ability to detect subtle structural and thermal variations provides a powerful tool for selecting and calibrating build parameters. For example, the observed orientation-dependent differences in strength suggest that designers can strategically align parts to exploit superior horizontal or transverse performance in load-bearing contexts. Likewise, insights into crystallinity and thermal transitions can guide the adjustment of laser power, scan speed, and layer thickness to promote stable microstructural development. Embedding FDA into SLS workflows thus reduces trial-and-error experimentation, shortens development cycles, and supports scalable quality control, contributing to more efficient and reliable additive manufacturing processes.

Future work may explore other semi-crystalline polymers processed by additive manufacturing to assess broader applicability. Real-time thermal monitoring during fabrication could improve understanding of phase changes. Expanding the functional data analysis to link mechanical, thermal, and structural properties may enhance predictive models. Long-term ageing and environmental exposure studies would help evaluate material durability in practical conditions.

## 5. Recommendations

It is recommended that these studies be extrapolated to other conditions to assess whether the FDA can be applied to more SLS conditions.

## Data Availability

Data are contained within the article.
